# Responses of renal hemodynamics and tubular functions to acute sodium–glucose cotransporter 2 inhibitor administration in non-diabetic anesthetized rats

**DOI:** 10.1038/s41598-017-09352-5

**Published:** 2017-08-25

**Authors:** Tuba M. Ansary, Yoshihide Fujisawa, Asadur Rahman, Daisuke Nakano, Hirofumi Hitomi, Hideki Kobara, Tsutomu Masaki, Jens M. Titze, Kento Kitada, Akira Nishiyama

**Affiliations:** 1 Department of Pharmacology, Faculty of Medicine, Kagawa University, Kagawa, Japan; 2 Life Science Research Center, Faculty of Medicine, Kagawa University, Kagawa, Japan; 3 Department of Gastroenterology and Neurology, Faculty of Medicine, Kagawa University, Kagawa, Japan; 4 Division of Clinical Pharmacology, Vanderbilt University School of Medicine, Nashville, TN USA

## Abstract

The aim of this study is to examine the effects of acute administration of luseogliflozin, the sodium–glucose cotransporter 2 (SGLT2) inhibitor, on renal hemodynamics and tubular functions in anesthetized non-diabetic Sprague Dawley (SD) rats and 5/6 nephrectomized (Nx) SD rats. Renal blood flow (RBF), mean arterial pressure (MAP), and heart rate (HR) were continuously measured and urine was collected directly from the left ureter. Intraperitoneal injection of luseogliflozin (0.9 mg kg^−1^) did not change MAP, HR, RBF, or creatinine clearance (CrCl) in SD rats (*n* = 7). Luseogliflozin significantly increased urine volume, which was associated with significantly increased urinary glucose excretion rates (*P* < 0.001). Similarly, luseogliflozin significantly increased urinary sodium excretion (from 0.07 ± 0.01 µmol min^−1^ at baseline to 0.76 ± 0.08 µmol min^−1^ at 120 min; *P* < 0.001). Furthermore, luseogliflozin resulted in significantly increased urinary pH (*P* < 0.001) and decreased urinary osmolality and urea concentration (*P* < 0.001) in SD rats. Similarly, in Nx SD rats (*n* = 5–6), luseogliflozin significantly increased urine volume and urinary glucose excretion (*P* < 0.001) without altering MAP, HR, RBF, or CrCl. Luseogliflozin did not elicit any significant effects on the other urinary parameters in Nx SD rats. These data indicate that SGLT2 inhibitor elicits direct tubular effects in non-diabetic rats with normal renal functions.

## Introduction

To manage type 2 diabetes mellitus, blockade of glucose reabsorption at the proximal tubule using sodium–glucose cotransporter 2 (SGLT2) inhibitors have been recently applied^1, 2^. SGLT2 is primarily expressed in the brush border membrane at S1 segments of the proximal convoluted tubule^3^. It has been suggested that approximately 90% of filtered glucose is reabsorbed by SGLT2^4^. Therefore, inhibition of this transporter leads to glycosuria, and selective SGLT2 inhibitors are considered therapeutic tools for treating type 2 diabetes. A review article reported that SGLT2 inhibition reduces blood pressure^5^; however, the mechanism responsible for SGLT2 inhibitor-induced blood pressure reduction is unclear. We have recently reported that reductions in blood pressure using SGLT2 inhibitors are associated with natriuresis in metabolic syndrome^6, 7^. Similarly, a clinical study reported that treatment with canagliflozin, the SGLT2 inhibitor, significantly increased urinary sodium excretion in patients with type 2 diabetes^8^. These findings suggest that SGLT2 inhibitor-induced blood pressure reduction is accompanied by natriuresis in patients with metabolic syndrome and diabetes.

Genetic knockout of SGLT2 resulted in increased urine flow and glucosuria without affecting the glomerular filtration rate (GFR) in mice^9^, and chronic treatment with an SGLT2 inhibitor showed sustained increases in urinary glucose excretion in wild-type mice^10^. Additionally, in a micropuncture study, it was found that acute injection of an SGLT2 inhibitor increased urine flow, urinary glucose, and sodium excretion in a rat model of early diabetes^11^. However, the effects of acute administration of SGLT2 inhibitors have not been examined in non-diabetic subjects. It is of particular importance that the pharmacological effects of SGLT2 inhibitors in non-diabetic subjects are studied to minimize any indirect influence induced by changes in blood glucose levels. In this regard, a study with pooled urine reported that treatment with an SGLT2 inhibitor increased the 24-h urine volume, urinary glucose, and sodium excretion in non-diabetic mice^12^. Similarly, an SGLT2 inhibitor tended to increase 24-h urinary excretion of sodium, potassium, and chloride in dogs^13^. However, it is difficult to exclude the possibility that urinary sodium excretion was influenced by sodium intake in these urine-storing studies. Thus, it remains unclear whether SGLT2 inhibitor-induced urinary changes are actually mediated by its direct tubular action or by other indirect mechanisms.

In the current study, we investigated the direct effects of SGLT2 inhibition on renal hemodynamics and tubular functions *in vivo* by examining the acute effects of luseogliflozin, the selective SGLT2 inhibitor^1, 3^, in anesthetized non-diabetic rats with normal kidney function. Clinically, side effects of SGLT2 inhibitors, such as polyuria and polydipsia, have been reported during the early stages of treatment^1, 2^, which leads to the restriction of use of an SGLT2 inhibitor, particularly in patients with chronic kidney disease (CKD). Therefore, we also examined the effects of a SGLT2 inhibitor on renal hemodynamics and functions in 5/6 nephrectomized (Nx) rat, a model of CKD.

## Results

**Figure 1:**
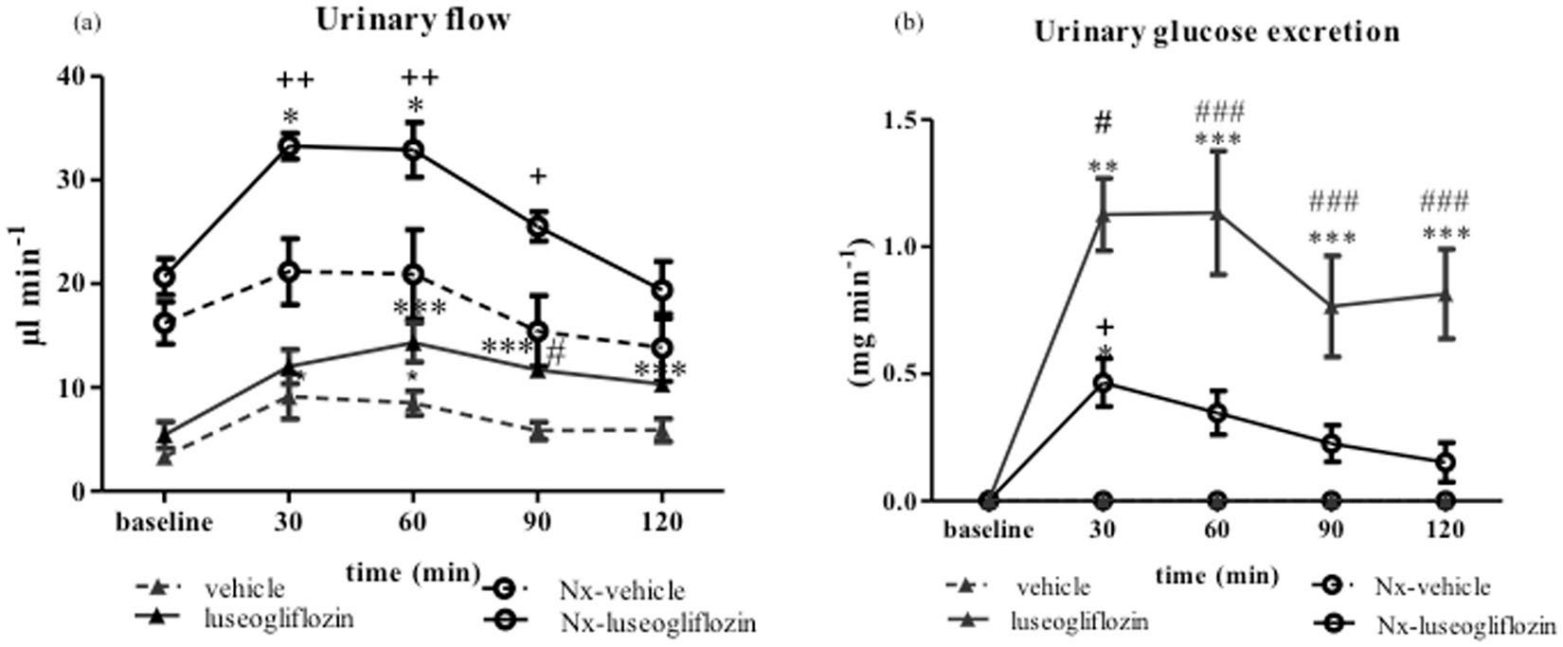
Urinary flow and glucose excretion in Protocol 1. Intravenous injection of luseogliflozin significantly increased urine flow (**a**) and glucose excretion (**b**) in both SD and Nx SD rats. SD, Sprague Dawley; Nx, 5/6 nephrectomized; vehicle, SD rats treated with vehicle; luseogliflozin, SD rats treated with luseogliflozin; Nx-vehicle, 5/6 Nx SD rats treated with vehicle; Nx-luseogliflozin, 5/6 Nx SD rats treated with luseogliflozin. Values are mean ± SEM. *P < 0.05, **P < 0.01  and ***P < 0.001 *vs*. baseline, ^*#*^ *P* < 0.05 and ^###^ *P* < 0.001 *vs*. vehicle, ^+^ *P* < 0.05 and ^++^ *P* < 0.01 *vs*. Nx*-*vehicle.

**Figure 2:**
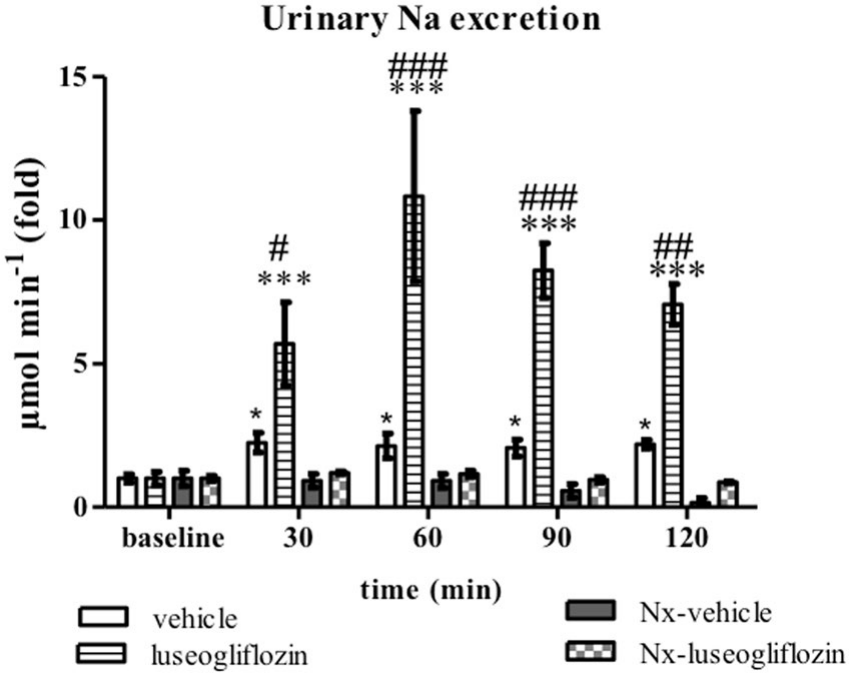
Urinary sodium excretion in Protocol 1. Intravenous injection of luseogliflozin significantly increased urinary sodium excretion in SD rats. SD, Sprague Dawley; Nx, 5/6 nephrectomized; vehicle, SD rats treated with vehicle; luseogliflozin, SD rats treated with luseogliflozin; Nx-vehicle, 5/6 Nx SD rats treated with vehicle; Nx-luseogliflozin, 5/6 Nx SD rats treated with luseogliflozin. Values are mean ± SEM. *P < 0.05 and ***P < 0.001 *vs*. baseline, ^*#*^ *P* < 0.05, ^##^ *P* < 0.01 and ^###^ *P* < 0.001 *vs*. vehicle.

A major limitation of these studies with intravenous infusion of luseogliflozin was that intravenously administered vehicle (2-hydroxylpropyl-β-cyclodextrin (HP-β-CD)) had considerable effects on urinary parameters. Specifically, vehicle significantly increased urine flow from 3.27 ± 0.57 to 8.47 ± 1.17 µl min^−1^ and urinary sodium excretion from 0.20 ± 0.02 to 0.44 ± 0.03 µmol min^−1^ after 60 minutes in Sprague Dawley (SD) rats (Figs 1 and 2). To minimize the effects of vehicle, preliminary experiments with intraperitoneal injection of luseogliflozin were performed. We found that intraperitoneal administration of vehicle did not change urine flow or urinary sodium excretion. Based on these findings, further experiments using intraperitoneal injection of luseogliflozin were also performed.

### Effects of intravenous injection of the SGLT2 inhibitor

#### Renal hemodynamics

Intravenous injection of luseogliflozin did not change mean arterial pressure (MAP), renal blood flow (RBF), heart rate (HR), or creatinine clearance (CrCl) in either SD or Nx SD rats (Table 1).

**Table 1:** Renal hemodynamics data of Protocol 1.

		vehicle	luseogliflozin	Nx -vehicle	Nx-luseogliflozin
MAP (mmHg)	baseline	103 ± 2	98 ± 3	104 ± 2	105 ± 1
	15	103 ± 3	99 ± 3	106 ± 5	113 ± 2
	45	108 ± 2	98 ± 3	102 ± 7	111 ± 4
	75	104 ± 2	97 ± 3	103 ± 3	107 ± 2
	105	104 ± 3	97 ± 2	104 ± 2	100 ± 4
	120	105 ± 3	99 ± 6	96 ± 4	97 ± 3
HR (beats min^−1^)	baseline	390 ± 8	386 ± 14	373 ± 18	339 ± 13
	15	384 ± 9	385 ± 13	372 ± 25	340 ± 11
	45	417 ± 8	400 ± 8	391 ± 11	358 ± 10
	75	427 ± 7	388 ± 13	381 ± 10	368 ± 13
	105	413 ± 10	369 ± 14	397 ± 16	367 ± 11
	120	418 ± 16	380 ± 10	390 ± 9	381 ± 9
Mean RBF (fold)	baseline	1.00 ± 0.02	1.00 ± 0.04	1.00 ± 0.12	1.00 ± 0.05
	5	0.91 ± 0.01	1.05 ± 0.05	1.00 ± 0.11	0.81 ± 0.11
	10	0.92 ± 0.01	0.96 ± 0.03	1.00 ± 0.11	1.00 ± 0.03
	15	1.03 ± 0.04	1.05 ± 0.05	0.97 ± 0.13	1.01 ± 0.04
	30	1.12 ± 0.13	0.98 ± 0.06	0.94 ± 0.08	1.02 ± 0.06
	60	1.08 ± 0.04	1.00 ± 0.03	0.87 ± 0.06	0.99 ± 0.07
	90	1.11 ± 0.02	0.99 ± 0.04	0.85 ± 0.03	0.89 ± 0.11
	120	1.08 ± 0.05	1.04 ± 0.04	0.81 ± 0.03	0.83 ± 0.14
CrCl (µl min^−1^ g^−1^ body weight)	baseline	3.22 ± 0.56	3.32 ± 0.71	5.24 ± 0.40	5.41 ± 0.42
	30	3.99 ± 0.45	4.10 ± 0.54	5.54 ± 0.42	5.97 ± 0.26
	60	3.76 ± 0.25	4.24 ± 0.63	5.48 ± 0.30	5.64 ± 0.29
	90	3.66 ± 0.21	4.14 ± 0.70	5.15 ± 0.40	5.22 ± 0.28
	120	3.75 ± 0.32	4.21 ± 0.76	4.94 ± 0.60	4.91 ± 0.33

MAP, mean blood pressure; HR, heart rate; RBF, renal blood flow; CrCl, creatinine clearance; SD, Sprague-Dawly; Nx, 5/6 nephrectomized SD rats; vehicle, SD rats treated with vehicle; luseogliflozin, SD rats treated with luseogliflozin; Nx-vehicle, 5/6 nephrectomized SD rats treated with vehicle; Nx-luseogliflozin, 5/6 nephrectomized SD rats treated with luseogliflozin. Values are mean ± SEM.

#### Urine flow and urinary glucose excretion

In SD rats, luseogliflozin significantly increased urine flow from 5.40 ± 1.27 µl min^−1^ at baseline to 10.33 ± 0.73 µl min^−1^ at 120 min (*P* < 0.001; Fig. 1a), which was associated with a significant increase in the urinary glucose excretion rate from 0.003 ± 0.001 mg min^−1^ at baseline to 0.81 ± 0.18 mg min^−1^ at 120 min (*P* < 0.001; Fig. 1b). These changes induced by luseogliflozin were significantly greater than those induced by vehicle (Fig. 1a and b). On the other hand, luseogliflozin-induced changes in glucose excretion were significantly less in Nx SD rats compared with SD rats (Fig. 1b).

#### Urinary excretion of sodium

In SD rats, luseogliflozin increased urinary sodium excretion from 0.34 ± 0.08 µmol min^−1^ at baseline to 2.41 ± 0.24 µmol min^−1^ at 120 min, by approximately seven-fold (*P* < 0.001). These changes induced by luseogliflozin were significantly greater than those induced by vehicle (Fig. 2). On the other hand, intravenous infusion of luseogliflozin did not have an acute effect on urinary sodium excretion in Nx SD rats (from 0.42 ± 0.04 µmol min^−1^ at baseline to 0.37 ± 0.01 µmol min^−1^ at 120 min) (Fig. 2).

### Effects of intraperitoneal injection of the SGLT2 inhibitor

#### Renal hemodynamics and blood glucose

Intraperitoneal injection of vehicle or luseogliflozin did not change MAP, RBF, HR, or CrCl in either SD or Nx SD rats (Table 2). Intraperitoneal injection of luseogliflozin did not significantly change plasma glucose level (from 142 ± 3 mg dl^−1^ at baseline to 147 ± 3 mg dl^−1^ at 120 min) in SD rats. Similarly, luseogliflozin did not significantly change plasma glucose level (from 131 ± 5 mg dl^−1^ at baseline to 133 ± 5 mg dl^−1^ at 120 min) in Nx SD rats.

**Table 2:** Renal hemodynamics data of Protocol 2.

		vehicle	luseogliflozin	Nx -vehicle	Nx-luseogliflozin
MAP (mmHg)	baseline	102 ± 2	101 ± 2	119 ± 3	123 ± 4
	15	106 ± 2	103 ± 2	123 ± 2	128 ± 4
	45	106 ± 2	104 ± 2	125 ± 3	128 ± 4
	75	105 ± 2	102 ± 2	127 ± 2	128 ± 3
	105	106 ± 2	103 ± 2	122 ± 2	131 ± 4
	120	109 ± 1	102 ± 1	119 ± 6	127 ± 5
HR (beat min^−1^)	baseline	385 ± 15	370 ± 15	386 ± 3	418 ± 7
	15	394 ± 15	376 ± 14	410 ± 14	398 ± 8
	45	397 ± 17	379 ± 12	380 ± 11	389 ± 7
	75	387 ± 16	378 ± 11	410 ± 17	373 ± 7
	105	398 ± 15	376 ± 10	372 ± 9	384 ± 8
	120	403 ± 2	378 ± 6	385 ± 9	388 ± 5
Mean RBF (fold)	baseline	1.00 ± 0.11	1.00 ± 0.09	1.00 ± 0.13	1.00 ± 0.18
	5	1.08 ± 0.10	1.06 ± 0.12	1.00 ± 0.13	1.04 ± 0.17
	10	1.06 ± 0.10	1.02 ± 0.10	1.01 ± 0.12	1.05 ± 0.18
	15	1.09 ± 0.11	1.02 ± 0.10	0.99 ± 0.12	1.03 ± 0.19
	30	1.09 ± 0.10	1.07 ± 0.11	0.97 ± 0.11	1.04 ± 0.18
	60	1.06 ± 0.11	1.06 ± 0.10	1.05 ± 0.11	1.07 ± 0.16
	90	1.10 ± 0.13	1.09 ± 0.10	1.07 ± 0.12	1.01 ± 0.14
	120	1.05 ± 0.12	1.19 ± 0.08	1.04 ± 0.13	1.07 ± 0.17
CrCl (µl min^−1^ g^−1^ body weight)	baseline	2.50 ± 0.16	2.49 ± 0.14	4.74 ± 0.16	4.73 ± 0.69
	30	2.77 ± 0.16	2.55 ± 0.15	5.08 ± 0.57	4.62 ± 0.67
	60	2.54 ± 0.19	2.98 ± 0.15	4.38 ± 0.17	4.68 ± 0.54
	90	2.60 ± 0.18	2.79 ± 0.26	4.61 ± 0.32	4.72 ± 0.43
	120	2.53 ± 0.27	2.76 ± 0.18	4.15 ± 0.21	4.62 ± 0.40

MAP, mean blood pressure; HR, heart rate; RBF, renal blood flow; CrCl, creatinine clearance; SD, Sprague-Dawly; Nx, 5/6 nephrectomized SD rats; vehicle, SD rats treated with vehicle; luseogliflozin, SD rats treated with luseogliflozin; Nx-vehicle, 5/6 nephrectomized SD rats treated with vehicle; Nx-luseogliflozin, 5/6 nephrectomized SD rats treated with luseogliflozin. Values are mean ± SEM.

#### Urine flow and urinary excretion of glucose

**Figure 3:**
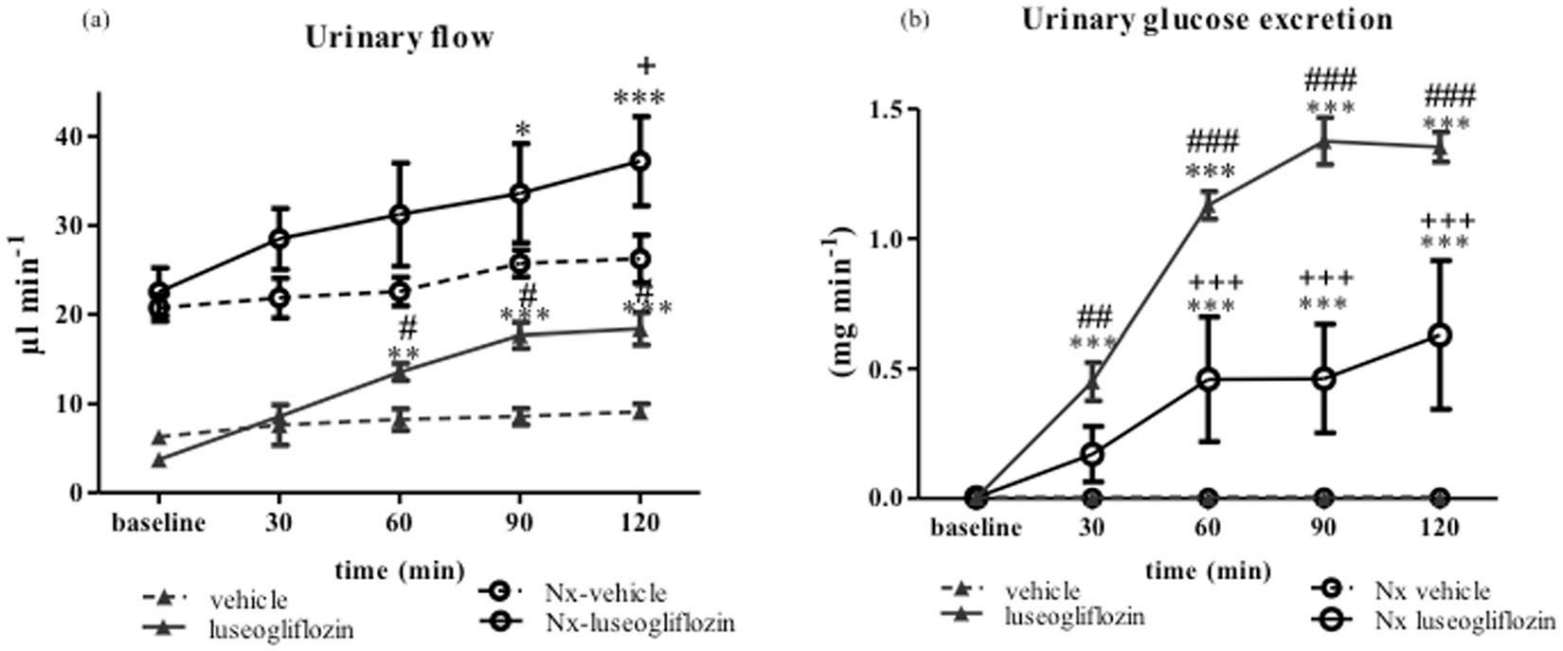
Urinary flow and glucose excretion in Protocol 2. Intraperitoneal injection of luseogliflozin significantly increased urinary flow (**a**) and glucose (**b**) excretion in both SD and Nx SD rats. SD, Sprague Dawley; Nx, 5/6 nephrectomized; vehicle, SD rats treated with vehicle; luseogliflozin, SD rats treated with luseogliflozin; Nx-vehicle, 5/6 Nx SD rats treated with vehicle; Nx-luseogliflozin, 5/6 Nx SD rats treated with luseogliflozin. Values are mean ± SEM. *P < 0.05, ** *P* < 0.01 and ***P < 0.001 *vs*. baseline, ^*#*^ *P* < 0.05, ^##^ *P* < 0.01 and ^###^ *P* < 0.001 *vs*. vehicle, ^+^ *P* < 0.05 and ^+++^ *P* < 0.001 *vs*. Nx*-*vehicle.

In SD rats, intraperitoneal injection of vehicle did not change any urinary parameters. However, intraperitoneal injection of luseogliflozin significantly increased urine flow from 3.71 ± 0.21 µl min^−1^ at baseline to 18.38 ± 1.8 µl min^−1^ at 120 min (*P* < 0.001; Fig. 3a), and was accompanied by a concomitant increase in urinary glucose excretion from 0.003 ± 0.001 mg min^−1^ at baseline to 1.35 ± 0.05 mg min^−1^ at 120 min (*P* < 0.001; Fig. 3b). Luseogliflozin-induced changes in urine flow and glucose excretion were significantly less in Nx SD rats compared with SD rats (Fig. 3a and b).

#### Urinary excretions of sodium

**Figure 4:**
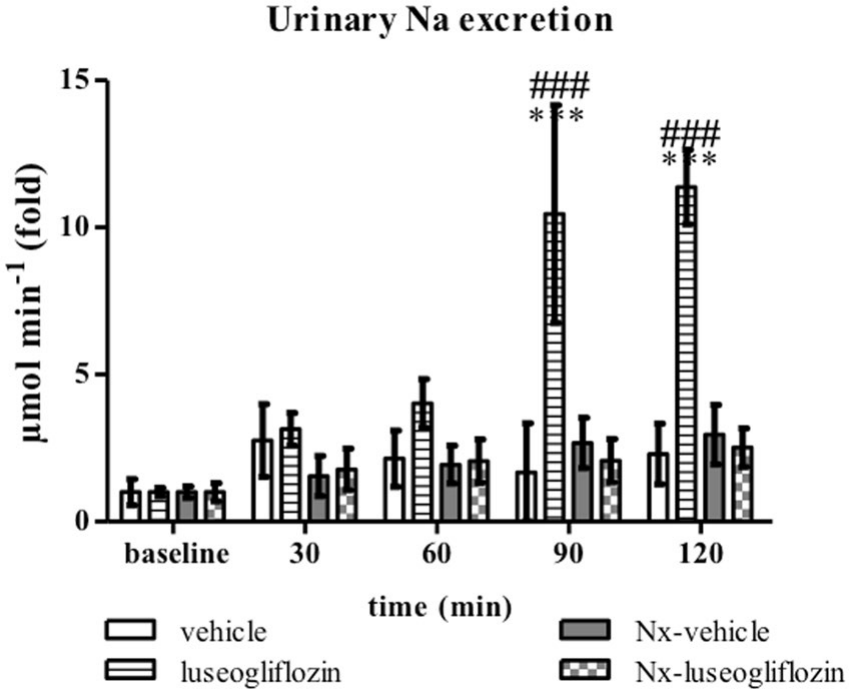
Urinary sodium excretion in Protocol 2. Intraperitoneal injection of luseogliflozin significantly increased urinary sodium excretion in SD rats. SD, Sprague Dawley; Nx, 5/6 nephrectomized; vehicle, SD rats treated with vehicle; luseogliflozin, SD rats treated with luseogliflozin; Nx-vehicle, 5/6 Nx SD rats treated with vehicle; Nx-luseogliflozin, 5/6 Nx SD rats treated with luseogliflozin. Values are mean ± SEM. ***P < 0.001 *vs*. baseline, ^###^ *P* < 0.001 *vs*. vehicle.

In SD rats, intraperitoneal administration of luseogliflozin induced an increase in urinary sodium excretion from 0.07 ± 0.01 µmol min^−1^ at baseline to 0.76 ± 0.08 µmol min^−1^ at 120 min, by approximately 10-fold (*P* < 0.001; Fig. 4). On the other hand, intraperitoneal administration of luseogliflozin did not have a significant effect on urinary sodium excretion in Nx SD rats (from 0.26 ± 0.15 µmol min^−1^ at baseline to 0.66 ± 0.17 µmol min^−1^ at 120 min; Fig. 4). Urinary potassium excretion was not significantly affected by acute intraperitoneal injection of luseogliflozin in either SD rats or Nx SD (data not shown).

#### Urinary pH and urea

**Figure 5:**
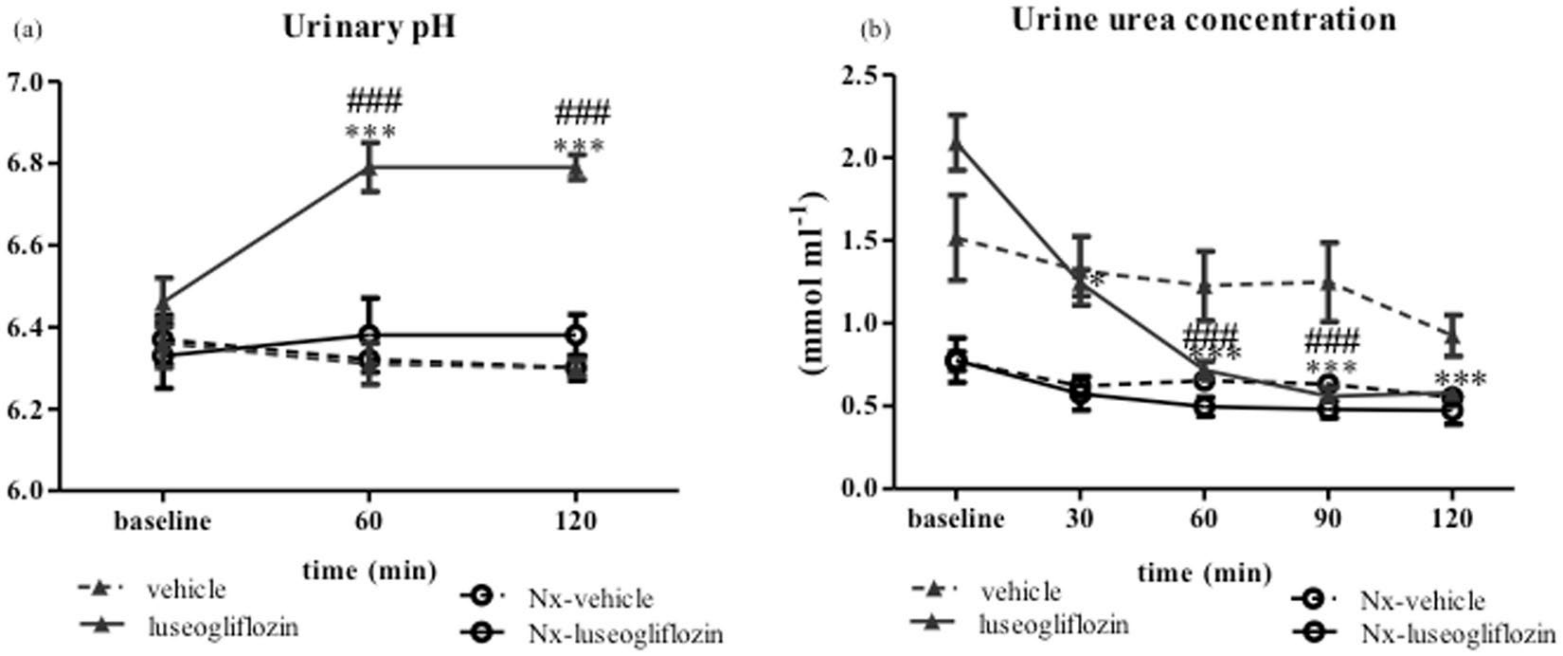
Urinary pH and urea concentration in Protocol 2. Effect of intraperitoneal injection of luseogliflozin on urine pH (**a**) and urea concentration (**b**). SD, Sprague Dawley; Nx, 5/6 nephrectomized; vehicle, SD rats treated with vehicle; luseogliflozin, SD rats treated with luseogliflozin; Nx-vehicle, 5/6 Nx SD rats treated with vehicle; Nx-luseogliflozin, 5/6 Nx SD rats treated with luseogliflozin. Values are mean ± SEM. *P < 0.05 and ***P < 0.001 *vs*. baseline, ^###^ *P* < 0.001 *vs*. vehicle.

In SD rats, luseogliflozin induced a significant increase in urinary pH from 6.53 ± 0.06 at baseline to 6.79 ± 0.03 at 120 min (*P* < 0.001; Fig. 5a), which was associated with significant reduction in urinary urea concentration from 2.09 ± 0.16 mmol ml^−1^ at baseline to 0.58 ± 0.04 mmol ml^−1^ at 120 min (*P* < 0.001; Fig. 5b).

#### Urinary osmolality and free water clearance

**Figure 6:**
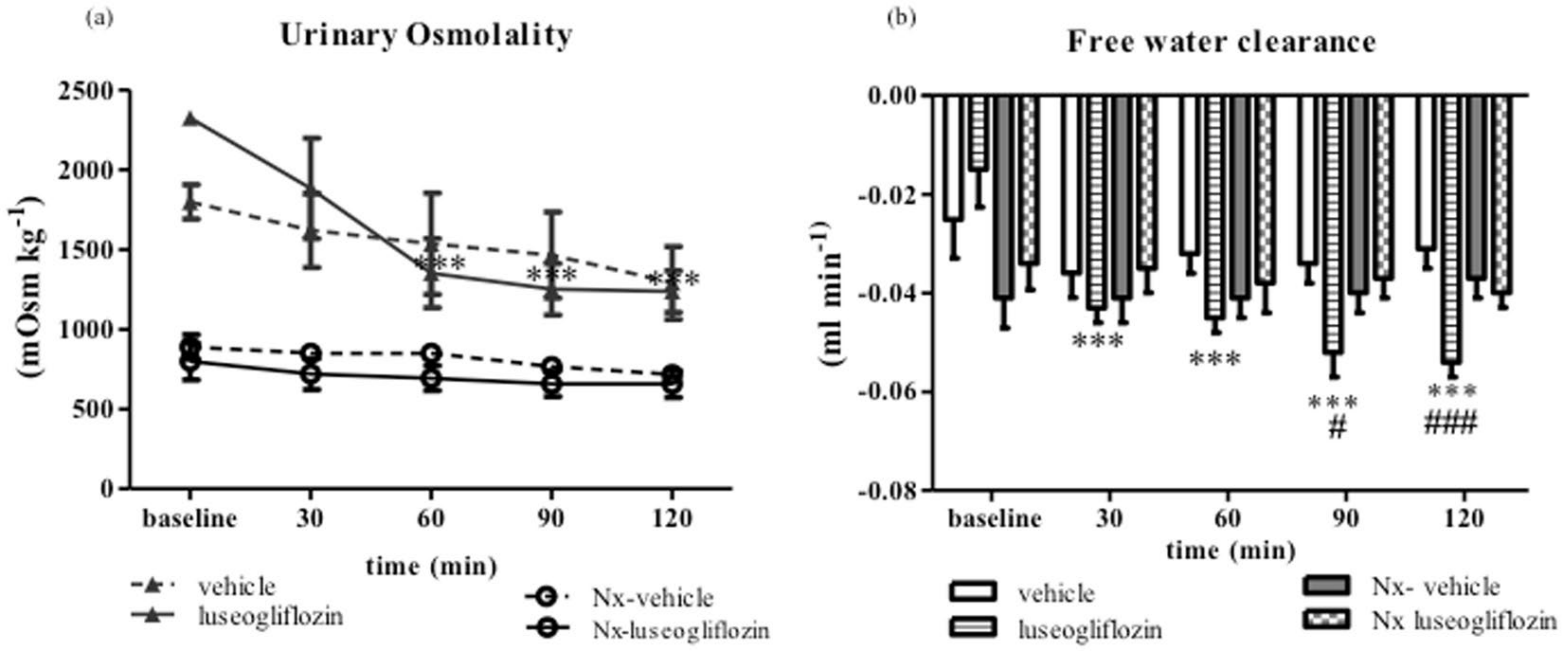
Urine osmolality and free water clearance in Protocol 2. Effect of intraperitoneal injection of luseogliflozin on urine osmolality (**a**) and free water clearance (**b**). SD, Sprague Dawley; Nx, 5/6 nephrectomized; vehicle, SD rats treated with vehicle; luseogliflozin, SD rats treated with luseogliflozin; Nx-vehicle, 5/6 Nx SD rats treated with vehicle; Nx-luseogliflozin, 5/6 Nx SD rats treated with luseogliflozin. Values are mean ± SEM. ***P < 0.001 *vs*. baseline, ^#^ *P* < 0.05 and ^###^ *P* < 0.001 *vs*. vehicle.

In SD rats, luseogliflozin generate a significant reductions in urinary osmolality from 2325 ± 44 mOsm kg^−1^ at baseline to 1236 ± 121 mOsm kg^−1^ at 120 min (*P* < 0.001; Fig. 6a). Accordingly, augmentation of calculated negative values of free water clearance were concomitant with these urinary changes induced by luseogliflozin in these rats (*P* < 0.001; Fig. 6b). Luseogliflozin did not have any significant effects on urinary pH, urea concentration, or osmolality in Nx SD rats.

### Effects of intraperitoneal injection of the SGLT2 inhibitor in inactin-anesthetized SD rats

We examined the effects of intraperitoneal administration of luseogliflozin in SD rats under inactin anesthesia (100 mg kg^−1^, i.p.; Protocol 3). Luseogliflozin significantly increased urinary glucose from 0.002 ± 0.0004 mg min^−1^ at baseline to 0.73 ± 0.07 mg min^−1^ at 120 min (*P* < 0.001). However, urinary sodium excretion was not significantly changed (from 0.09 ± 0.02 µmol min^−1^ at baseline to 0.11 ± 0.04 µmol min^−1^ at 120 min). Other renal parameter results in Protocol 3 are shown in Supplementary Figures 1–3.

## Discussion

Chronic treatment of a SGLT2 inhibitor elicits several systemic effects on blood glucose, body weight, blood pressure, etc. To avoid any systemic changes, the present study utilized acute renal clearance method to examine the effects of luseogliflozin on renal hemodynamics and tubular functions. We found that acute administration of an SGLT2 inhibitor induced natriuresis without changing renal hemodynamics in non-diabetic SD rats. In Protocol 1, rats were anesthetized with sodium pentobarbital and maintained with isoflurane, and luseogliflozin was injected intravenously. Luseogliflozin significantly increased urine flow and urinary glucose excretion in SD rats without affecting MAP, RBF, or CrCl. To avoid any possible effects from the vehicle, luseogliflozin was also injected intraperitoneally (Protocol 2). We confirmed that intraperitoneal injection of vehicle did not change any parameters. However, intraperitoneal injection of luseogliflozin resulted in substantial glycosuria and natriuresis without changing renal hemodynamics in SD rats. The findings that these acute tubular effects of the SGLT2 inhibitor were observed in non-diabetic rats strongly support the hypothesis that the SGLT2 inhibitor elicits direct tubular effects independently of blood-glucose changes.

Our data showed that acute administration of luseogliflozin did not have any effects on MAP, mean RBF, or CrCl in non-diabetic SD rats. Previous studies have reported that chronic SGLT2 inhibitor treatment reduced GFR in type 1 diabetic patients with glomerular hyperfiltration^14^ and in type 1 diabetic Akita mice^10^. Similarly, chronic SGLT2 blockade significantly decreased GFR in type 2 diabetic rats^1, 15^ and patients^16, 17^. These studies have indicated that SGLT2 inhibitor-induced reduction in GFR is associated with an increase in the activity of tubuloglomerular feedback (TGF) mechanism. However, SGLT2 inhibitor did not have a significant effect on GFR in the early diabetic condition^18^. In agreement with the results in non-diabetic mice^9^ and rats^19^, our data also showed that acute infusion of luseogliflozin did not have any immediate significant effects on renal hemodynamics in non-diabetic SD rats. Thus, it seems that SGLT2 inhibitor-induced renal hemodynamic changes are significant in diabetes and not obvious in non-diabetes. Less amount of glucose reabsorption with SGLT2 inhibition may be related to less effect of SGLT inhibition on the activation of TGF mechanism^11^.

Acute administration of luseogliflozin significantly increased urine flow and urinary sodium excretion without affecting renal hemodynamics in non-diabetic SD rats. These acute responses are consistent with a previous study in which it was reported that luseogliflozin increased urinary sodium excretion 3 days after its administration in metabolic syndrome rats^6^. A clinical study has also shown that canagliflozin, a SGLT2 inhibitor, significantly increased urine volume and urinary sodium excretion in patients with type 2 diabetes^8^. As previously noted, a micropuncture study reported that an SGLT2 inhibitor increased urinary sodium excretion in diabetic rats^11^. Collectively, these data suggest that SGLT2 inhibitor elicits direct tubular effects in both diabetic and non-diabetic subjects.

In the current study, we expected urinary osmolality to increase following treatment with the SGLT2 inhibitor owing to a significant urinary glucose excretion. However, acute administration of the SGLT2 inhibitor consistently reduced urinary osmolality in SD rats (*P* < 0.001). We also observed that the urea concentration significantly decreased with the SGLT2 inhibitor (*P* < 0.001) in SD rats. As suggested previously^20^, high urinary glucose retains water in the tubular lumen, which dilutes urinary urea. Further studies are needed to examine the effects of SGLT2 inhibitors on the activities of water and urea transporters.

In this study, we also examined the effects of luseogliflozin in Nx SD rats, a model of CKD. In agreement with previous findings^21^, MAP elevation was observed in anesthetized Nx SD rats. Studies have indicated that in the remnant kidney model, chronic reduction in renal mass leads to an increase in blood pressure^22, 23^. In these animals, glomerular hyperfiltration of the remaining nephrons was observed, probably to compensate for nephron loss^24^ and following structural damage, and the kidney may have allowed an increase in the GFR for adaptation^25^. However, acute administration of the SGLT2 inhibitor did not alter RBF or CrCl in Nx SD rats. These data are inconsistent with a previous clinical study in which SGLT2 inhibitor significantly decreased the GFR in type 1 diabetic patients with glomerular hyperfiltration^14^. Therefore, it is possible that SGLT2 inhibitor-induced changes in renal hemodynamics are not obvious in non-diabetics with glomerular hyperfiltration. Alternatively, the possibility cannot be excluded that responses in the GFR to SGLT2 inhibitors are species specific.

When compared with SD rats, rats after 2 weeks of 5/6 nephrectomy displayed a marked increase in urine volume at baseline. Previous studies have indicated that increases in urine volume were associated with downregulation of the water channels in the collecting duct and proximal tubule^26^. Intraperitoneal administration of luseogliflozin induced a significant increase in urine output in Nx SD rats (*P* < 0.001). In agreement with previous studies^24^, after administering luseogliflozin, Nx SD rats exhibited lower glucose excretion compared with the SD rats, probably due to renal functional impairments^27^. In agreement with previous studies^28–30^, Nx SD rats showed greater urinary sodium and potassium excretion compared with SD rats. However, acute blockade of SGLT2 by luseogliflozin did not alter urinary sodium excretion in Nx SD rats. We have no good explanation as to why the SGLT2 inhibitor does not increase urinary sodium excretion in 5/6 Nx SD rats. One possibility is that reduced renal mass diminishes the functional activities of the sodium transporters, as shown by previous studies^28, 31, 32^. Consistent with a previous study^33^, we observed a significant reduction in the ability of urine to be concentrated and the urinary urea concentration in Nx SD rats. Luseogliflozin treatment similarly did not result in further reductions in urine osmolality or urea concentration in Nx SD rats. Interestingly, we found a significant increase in urinary pH in SD rats after luseogliflozin administration (*P* < 0.001), which was not observed in Nx SD rats. These results are consistent with a previous study in which systemic administration of phlorizin (a non-selective SGLT inhibitor) resulted in bicarbonaturia^34^. However, precise mechanism responsible for tubular effect of SGLT2 inhibitor is not clear, due to the limitations of *in vivo* clearance studies. Further studies are needed to explore the molecular mechanisms for tubular effects of SGLT2 inhibitor.

The major challenge in this study was the solubility of luseogliflozin. In this study, we used dimethyl sulfoxide (DMSO) and HP-β-CD as vehicle. Luseogliflozin could be dissolved in only 100% DMSO, but acute administration of high concentration DMSO caused hematuria. Intravenous administration of HP-β-CD caused neither hematuria nor renal hemodynamic changes; however, urinary parameters were significantly affected by administration of HP-β-CD. On the other hand, we confirmed that intraperitoneal administration of HP-β-CD showed minimal effect on systemic and renal parameter. Preliminary experiments showed that 0.9 mg kg^−1^ is the maximum dose of luseogliflozin and the minimum concentration of HP-β-CD is 4.5%. We have also documented that intraperitoneal administration of luseogliflozin at 0.9 mg kg^−1^ caused significant glucosuria, whereas 4.5% HP-β-CD did not change any renal parameters. Another crucial point is the application of an adequate anesthetic. In Protocol 3, we administered luseogliflozin in SD rats under inactin anesthesia. However, the predicted effect on urinary sodium excretion by luseogliflozin was absent, possibly because of the effects of inactin. Previous studies have clearly shown that inactin has a strong ability to inactivate urinary sodium excretion^35^. For these reasons, we used sodium pentobarbital and isoflurane as anesthetics in the current study.

In conclusion, acute administration of the SGLT2 inhibitor induced significant increases in urinary glucose and sodium excretion without altering renal hemodynamics in non-diabetic SD rats. These renal effects of the SGLT2 inhibitor were markedly attenuated in 5/6 Nx SD rats. These findings support the hypothesis that SGLT2 inhibitors elicit direct tubular effects without changes in plasma glucose levels.

## Methods

### Animals

All experimental procedures were performed according to the guidelines for the care and use of animals established by Kagawa University (Kagawa, Japan). Male SD rats (Japan SLC, Inc., Shizuoka, Japan) were housed in specific pathogen-free animal facilities at a controlled temperature (24 ± 2 °C) and humidity (55 ± 5%) on a 12-h light–dark cycle. Animals had free access to standard chow and water. All surgical and experimental procedures were approved by the Animal Care and Use Committee, Kagawa University.

By following the excision remnant kidney model^22^, an experimental Nx rat model was induced by ablation of two thirds of the left kidney at 7 weeks of age and, after a further week, a total right nephrectomy was performed. After a 2-week acclimation period, rats were assigned to one of two groups: Nx-luseogliflozin and Nx-vehicle.

### Experimental procedures

SD and Nx SD rats were anesthetized with sodium pentobarbital (50 mg kg^−1^, i.p.) and isoflurane (0.5–1.5% in air) or inactin (100 mg kg^−1^, i.p.) at 9–11 weeks of age. Then, animals were placed on a heated pad to maintain body temperature at 37 °C. A polyethylene catheter (PE-60; Becton Dickinson and Company, Sparks, MD, USA) was inserted into the abdominal aorta via the right femoral artery for blood pressure measurement and collection of arterial blood. We collected urinary samples for every 30 minutes. The left kidney was exposed through a retroperitoneal flank incision. The renal artery was carefully isolated from the tissue connecting the renal hilum cephalic. A Doppler flow probe (HDP 10.20 R; Crystal Biotech, Northborough, MA, USA) was placed around the renal artery and RBF was continuously monitored. A polyethylene catheter (tapered PE-50; Becton Dickinson and Company) was inserted into the left ureter for urine collection. After surgery, each rat was kept isolated for 60 min to allow for stabilization of MAP, RBF, and urine flow. All rats were given saline as a maintenance fluid at a dose of 0.7 ml h^−1^. Blood was collected in EDTA- containing tubes. At the end of experiments, animals were euthanized by overdose of pentobarbital (250 mg kg^−1^).

### Experimental protocols

Full details of experimental protocols are given in Supplementary Figures 4 and 5.

#### Protocol 1

SD and Nx SD rats were anesthetized with pentobarbital and isoflurane, and randomly assigned to one of four groups (n = 5–7 for each): Group 1, SD rats administered luseogliflozin; Group 2, SD rats administered vehicle (HP-β-CD at 4.5%); Group 3, Nx SD rats administered luseogliflozin; and Group 4, Nx SD rats administered vehicle. Luseogliflozin (0.3 mg kg^−1^) or vehicle at 0.1 ml was administered intravenously. Preliminary experiments showed that intravenous injection of luseogliflozin (0.3 mg kg^−1^) significantly increased urinary glucose excretion.

#### Protocol 2

SD and Nx SD rats were anesthetized with pentobarbital and isoflurane, and randomly assigned to one of four groups (n = 5–7 for each): Group 1, SD rats administered luseogliflozin; Group 2, SD rats administered vehicle; Group 3, Nx SD rats administered luseogliflozin; and Group 4, Nx SD rats administered vehicle. Luseogliflozin (0.9 mg kg^−1^) or vehicle at 0.1 ml was administered intraperitoneally. Preliminary studies showed that the minimum dose of HP-β-CD was 4.5% in 0.1 ml to dissolve 0.9 mg kg^−1^ luseogliflozin.

#### Protocol 3

SD rats were anesthetized with inactin and assigned to one of two groups (n = 7 for each): Group 1, SD rats administered luseogliflozin; and Group 2, SD rats administered vehicle. Luseogliflozin (0.9 mg kg^−1^) or vehicle was administered at 0.1 ml intraperitoneally.

### Electrolyte and other analyses

Plasma and urine electrolytes (Na^+^ and K^+^) were determined by flame photometry (EFOX 5053; Eppendorf, Burladingen, Germany). Plasma and urinary glucose levels were measured using an automated analyzer (7020 Automatic Analyzer; Hitachi High-Technologies, Tokyo, Japan). Urinary and plasma levels of creatinine (LabAssay^TM^ creatinine, Wako Pure Chemical Industries, Osaka, Japan), urea (urea assay kit; Biovision, Milpitas, CA, USA), and blood urea nitrogen (Urea N B, Urease-Indophenol method, Wako) were determined using commercially available assay kits. Urine and plasma osmolality were measured by vapor pressure osmometry (Wescor, Logan, UT, USA). Urinary pH was determined using PEHANON pH-test strips (Macherey-Nagel, Düren, Germany).

### Statistical analysis

Data are mean ± standard error of the mean (SEM). The statistical significance of difference was determined using two-way analysis of variance followed by Bonferroni post-tests using GraphPad Prism software ver. 5.0 (GraphPad, La Jolla, CA, USA). A *P*-value < 0.05 was considered statistically significant.

### Materials

Luseogliflozin, a selective SGLT2-inhibitor, was provided by Taisho Pharmaceutical Co. (Tokyo, Japan)^1, 3^. HP-β-CD was purchased from Wako (Osaka, Japan).

## Electronic supplementary material

Supplementary information
