# Supplementary material for: Responses of renal hemodynamics and tubular functions to acute sodium–glucose cotransporter 2 inhibitor administration in non-diabetic anesthetized rats
Source: Sci Rep. 2017 Aug 25;7:9555. doi: 10.1038/s41598-017-09352-5 (PMC5572725; doi:10.1038/s41598-017-09352-5)

Supplementary information

Responses of renal hemodynamics and tubular functions to acute sodium–glucose cotransporter 2 inhibitor administration in non-diabetic anesthetized rats

Tuba M. Ansary[1], Yoshihide Fujisawa[2], Asadur Rahman[1], Daisuke Nakano[1], Hirofumi Hitomi[1], Hideki Kobara[3], Tsutomu Masaki[3], Jens M. Titze[4], Kento Kitada[1,4], Akira Nishiyama[1]

[1]Department of Pharmacology, Faculty of Medicine, Kagawa University, Kagawa, Japan

[2]Life Science Research Center, Faculty of Medicine, Kagawa University, Kagawa, Japan

[3]Department of Gastroenterology and Neurology, Faculty of Medicine, Kagawa University, Kagawa, Japan

[4]Division of Clinical Pharmacology, Vanderbilt University School of Medicine, Nashville, TN, USA

Corresponding author

Akira Nishiyama, MD, PhD

Department of Pharmacology, Faculty of Medicine, Kagawa University, 1750-1 Ikenobe, Miki-cho, Kita-gun, Kagawa 761-0793, Japan

Tel.: +81 87 891 2125

Fax: +81 87 891 2126

E-mail: akira@kms.ac.jp

SUPPLEMENTARY FIGURE LEGENDS

Supplementary Figure 1 Urinary flow and glucose excretion in Protocol 3.

Intraperitoneal injection of luseogliflozin significantly increased urinary flow (a) and glucose excretion (b) in SD rats. SD, Sprague Dawley; vehicle, SD rats treated with vehicle; luseogliflozin, SD rats treated with luseogliflozin. Values are mean ± SEM. *P<0.05 and ***P<0.001 *vs.* baseline, $^{\#}P$<0.05, $^{\#\#}$ P<0.01 and $^{\#\#\#}$ P<0.001 *vs.* vehicle.

Supplementary Figure 2 Urinary sodium and potassium excretion in Protocol 3

Luseogliflozin treatment did not change urinary sodium (a) or potassium (b). SD, Sprague Dawley; vehicle, SD rats treated with vehicle; luseogliflozin, SD rats treated with luseogliflozin. Values are mean ± SEM.

Supplementary Figure 3 Urinary pH (a), urea concentration (b), urine osmolality (c), and free water clearance (d) in Protocol 3

SD, Sprague Dawley; vehicle, SD rats treated with vehicle; luseogliflozin, SD rats treated with luseogliflozin. Values are mean ± SEM. **P*<0.05 and ****P*<0.001 *vs.* baseline, $^{\#}P$<0.05 and $^{\#\#\#}$ P<0.001 *vs.* vehicle.

Supplementary Figure 4 Diagram of the study design for Protocols 1 and 2.

After anesthetizing Sprague Dawley (SD) rats with sodium pentobarbital and isoflurane, urine and blood are obtained at baseline. Luseogliflozin is given by intravenous and intraperitoneal injection in Protocols 1 and 2, respectively. Then, urine is collected every 30 min and blood pressure, heart rate, and renal blood flow are measured every 15 min. At the end of the experiments, blood is taken again.

Supplementary Figure 5 Diagram of the study design for Protocol 3.

Sprague Dawley (SD) rats are anesthetized with inactin and the other experimental protocols are the same as before.

Supplementary Table 1. **Renal hemodynamics data of Protocol 3**

		vehicle	luseogliflozin
MAP (mmHg)	baseline	89 ± 2	98 ± 6
	15	92 ± 2	96 ± 4
	45	90 ± 2	94 ± 4
	75	91 ± 4	93 ± 5
	105	89 ± 2	92 ± 5
	120	89 ± 2	91 ± 4
HR (beat min^{-1})	baseline	354 ± 4	345 ± 9
	15	355 ± 9	355 ± 7
	45	349 ± 6	345 ± 5
	75	347 ± 5	344 ± 4
	105	347 ± 4	334 ± 4
	120	362 ± 2	328 ± 8
Mean RBF (fold)	baseline	1.00 ± 0.06	1.00 ± 0.04
	5	0.99 ± 0.06	1.04 ± 0.07
	10	1.00 ± 0.06	1.03 ± 0.07
	15	0.97 ± 0.04	1.00 ± 0.07
	30	0.99 ± 0.04	0.99 ± 0.06
	60	0.97 ± 0.04	1.02 ± 0.06
	90	0.97 ± 0.04	1.05 ± 0.08
	120	1.02 ± 0.03	1.03 ± 0.07
CrCl (μl min^{-1} g^{-1}	baseline	2.15 ± 0.37	2.23 ± 0.19
body weight)	30	2.71 ± 0.25	2.72 ± 0.15
	60	2.25 ± 0.18	2.68 ± 0.14
	90	2.31 ± 0.37	2.53 ± 0.09
	120	2.03 ± 0.36	2.66 ± 0.13

SD, Sprague-Dawly rats; MAP, mean blood pressure; HR, hear rate; RBF, renal blood flow; CrCl, creatinine clearance; vehicle, SD rats treated with vehicle; luseogliflozin, SD rats treated with luseogliflozin. Values are mean ± SEM.

Supplementary Figure 1.

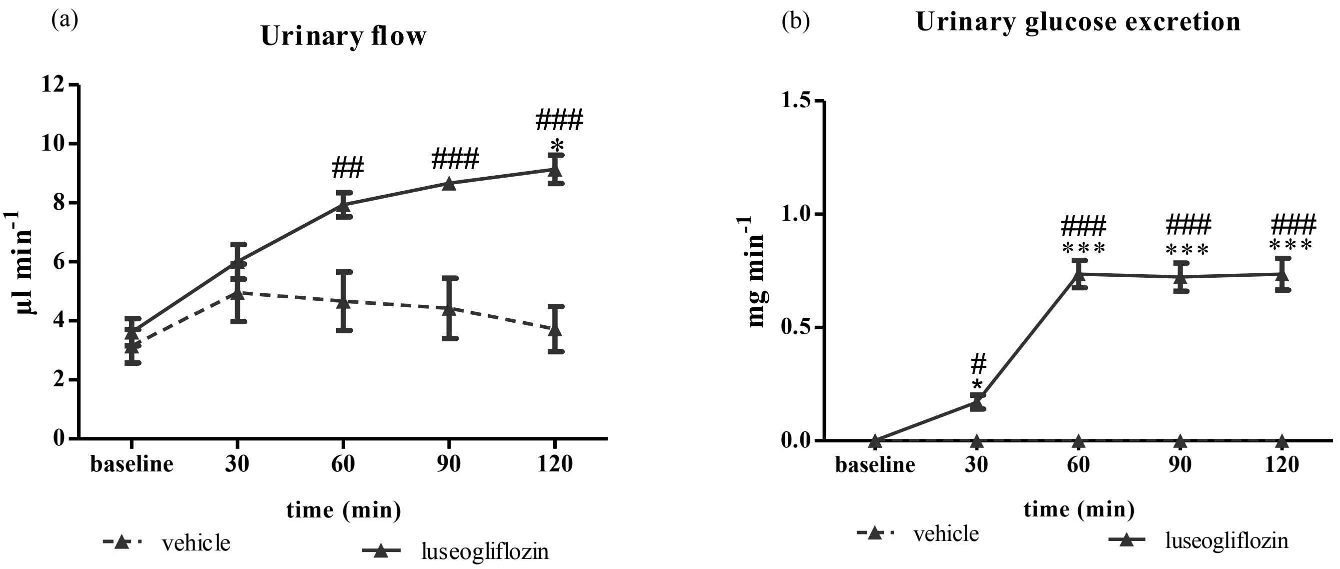

Supplementary Figure 2.

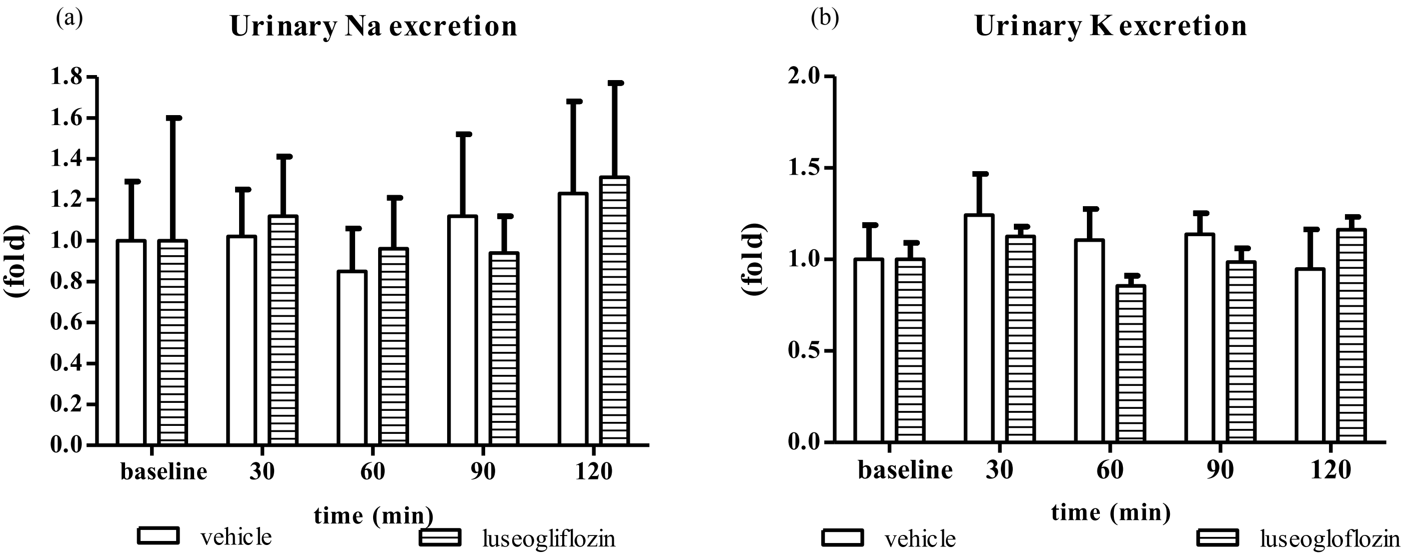

Supplementary Figure 3.

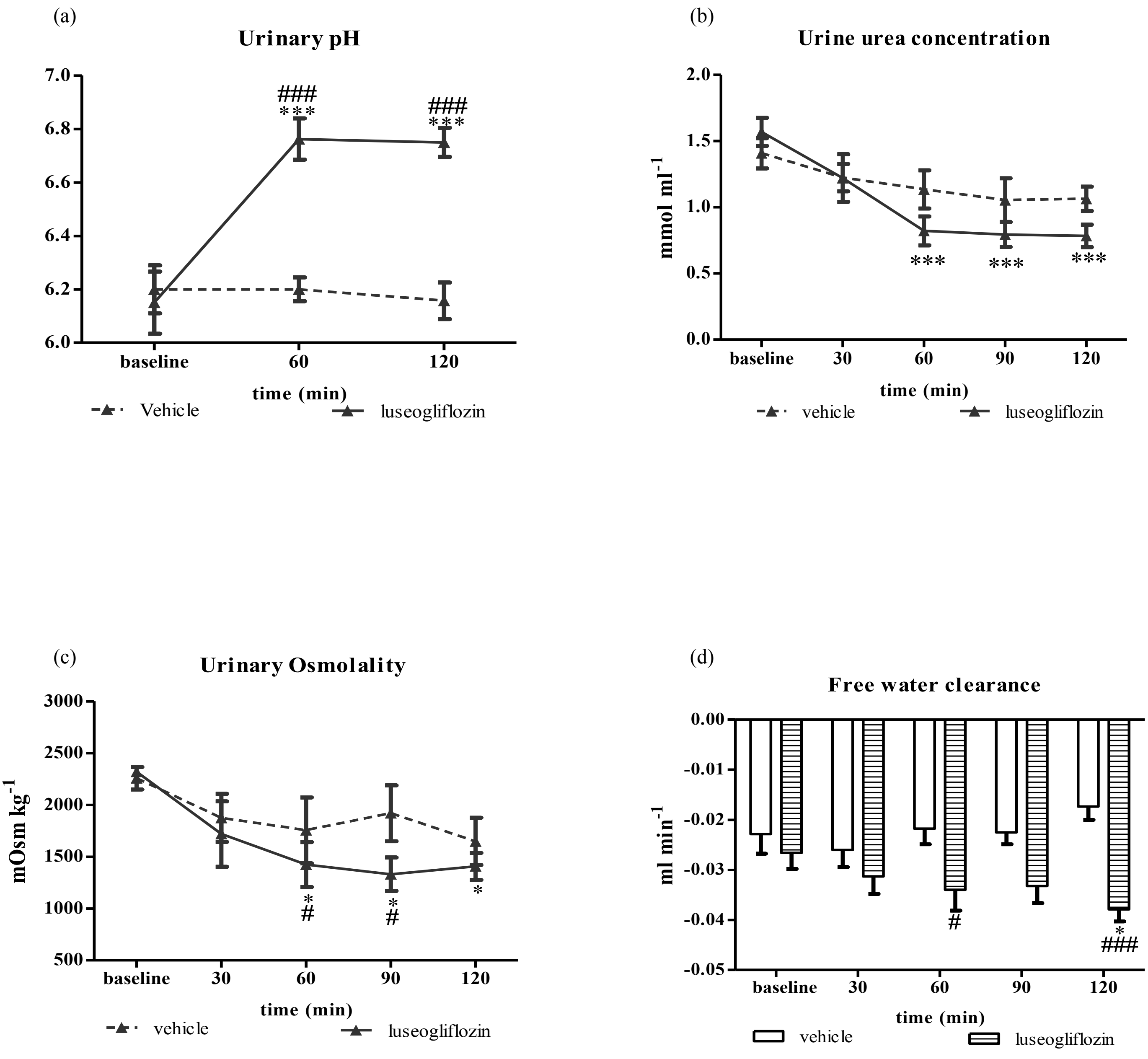

Supplementary Figure 4.

Protocol 1

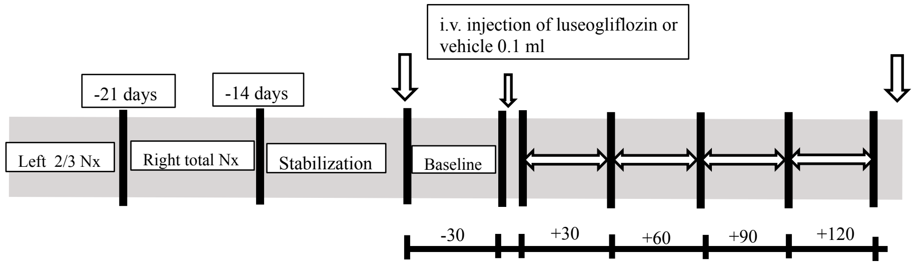

Protocol 2

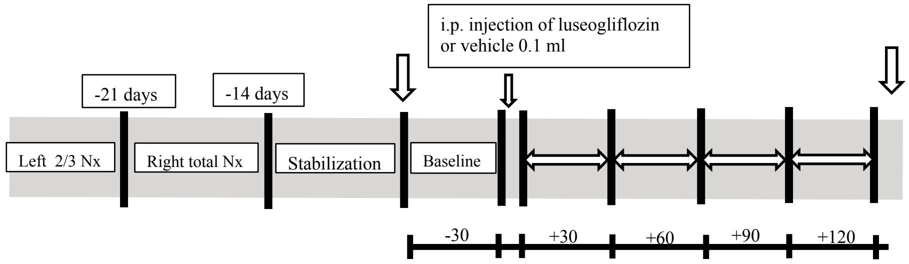

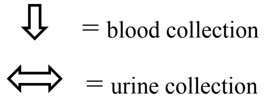

Supplementary Figure 5.

Protocol 3

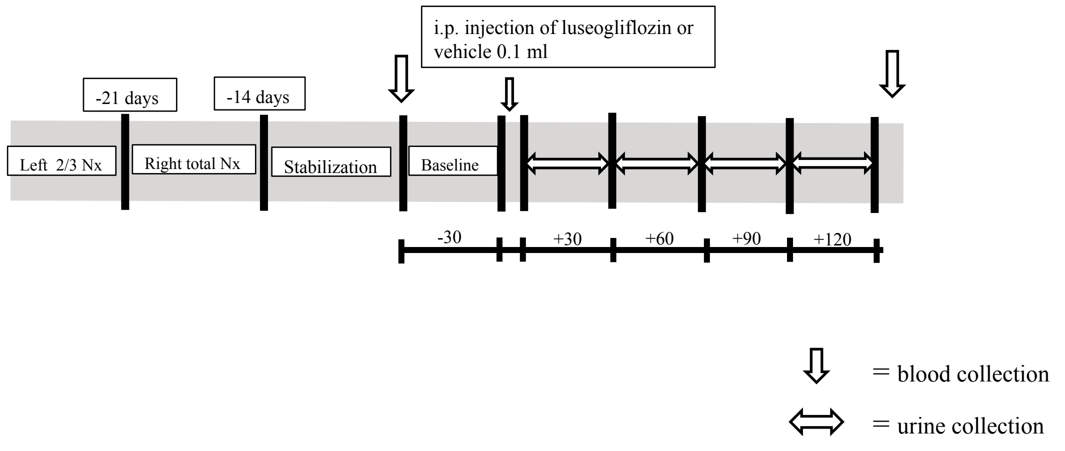

Supplement: Supplementary file 1 — Supplementary information [file 41598_2017_9352_MOESM1_ESM.pdf]
